# Patterns of Antimicrobial Resistance in the Causative Organisms of Spontaneous Bacterial Peritonitis: A Single Centre, Six-Year Experience of 1981 Samples

**DOI:** 10.1155/2014/917856

**Published:** 2014-03-20

**Authors:** Sara Sheikhbahaei, Alireza Abdollahi, Nima Hafezi-Nejad, Elham Zare

**Affiliations:** ^1^Department of Pathology, Imam Hospital Complex, Tehran University of Medical Sciences (TUMS), P.O. Box 14197-33141, Tehran, Iran; ^2^Students' Scientific Research Center (SSRC), Tehran University of Medical Sciences (TUMS), P.O. Box 14155-6537, Tehran, Iran

## Abstract

*Background/Aims*. Spontaneous bacterial peritonitis (SBP) is one of the leading causes of morbidity and mortality in patients with cirrhosis. This study aims to determine the microbial agents of SBP and the pattern of antibiotic resistance, in a large number of ascitic samples. *Methodology*. In a cross-sectional, single center, hospital based study, 1981 consecutive ascitic fluid samples were recruited from 2005 to 2011. Samples were dichotomized into three-year periods, in order to assess the trend of resistance to the first-line empirical antibiotics. *Results*. SBP was found in 482 (24.33%) of samples, of which 314 (65.15%) were culture positive. The most prevalent isolated pathogen was *E. coli* (33.8%), followed by *staphylococcus aureus* (8.9%) and *Enterococcus* (8.6%). No significant changes in the proportion of gram-negative/gram-positive infections occurred during this period. A percentage of resistant strains to cefotaxime (62.5%, 85.7%), ceftazidim (73%, 82.1%), ciprofloxacin (30, 59.8%), ofloxacin (36.8%, 50%), and oxacilin (35%, 51.6%) were significantly increased. *E. coli* was most sensitive to imipenem, piperacillin-tazobactam, amikacin, ceftizoxime, and gentamicin. *Conclusions*. The microbial aetiology of SBP remains relatively constant. However, the resistance rate especially to the first-line recommended antibiotics was significantly increased. This pattern must be watched closely and taken into account in empirical antibiotic treatment.

## 1. Introduction

Spontaneous bacterial peritonitis (SBP) is one of the leading causes of morbidity and mortality in patients with cirrhosis [1–3]. Unselected hospitalized cirrhotic patients with ascites were estimated to have 10%–30% risk of developing SBP [2, 3]. Early diagnosis and a prompt antibiotic therapy have considerably decreased the mortality rate associated with an episode of SBP from 80% to approximately 20–30% in the last decade [1, 2, 4–6].

SBP is defined as a monomicrobial infection of the ascitic fluid, which is not accompanied by a definite evidence of a surgically treatable origin [1, 3, 4]. The infection occurs following a translocation or haematogenous dissemination of the intestinal flora. Intestinal bacterial overgrowth can also exacerbate the condition [1, 3]. Studies have indicated that gram-negative Enterobacteriaceae such as* Escherichia coli* (*E. coli*) was the most common isolated organisms in SBP [1, 3, 7].

Diagnosis of SBP is established by an elevated ascitic fluid polymorphonuclear leukocyte (PMNL) count (≥250 cells/mm^3^) [1, 3, 4]. Some studies suggest that the type and the etiology of SBP have been changing in the recent years. Involvement with gram-positive bacteria and increased frequency of multiple antibiotic resistant bacteria are evidences that support this viewpoint [6, 8, 9].

Based on EASL guidelines, third-generation cephalosporins (including cefotaxime and ceftriaxone) are recommended as the first-line therapy [1–4]. However, knowledge about the local epidemiological pattern of antibiotic resistance would be necessary for an effective treatment [2]. According to the pattern of antibiotic consumption, great differences exist in antibiotic sensitivity and resistance among various countries. Meanwhile, information regarding the spectrum of the involved bacteria and the pattern of antibiotic resistance in developing countries is scarce. The present study aims to determine the current causative agents of SBP and the pattern of antibiotic resistance, in a large number of ascitic samples. The antibiotic susceptibility patterns are delineated by in vitro methods. We further assess the trends of resistance to the first-line empirical antibiotics within a six-year period. The result of this study could be implicated in future management and treatment of patients with SBP in similar settings.

## 2. Methodology

### 2.1. Samples

This cross-sectional hospital based study was conducted in Imam Hospital Complex affiliated to the Tehran University of Medical Sciences, Iran. All ascitic fluid samples, referred to the pathology division of the hospital from April 2005 to September 2011, were included. Samples were recruited from cirrhotic patients with new onset grade 2 or 3 ascitic patients hospitalized for worsening of ascites and patients who developed symptoms suggestive of SBP or any complication of cirrhosis [1].

The ascitic fluid analyses include cell counts and differential, culture, and antibiotic susceptibility pattern. Culture-positive SBP was diagnosed in the presence of ascitic fluid PMNL ≥ 250 cells/mm^3^ and positive ascitic fluid culture for a single organism. When the ascitic fluid culture results are negative, but the PMNL counts are 250 cells/mm^3^ or higher, culture-negative neutrocytic ascites were diagnosed [1, 10]. Samples with polymicrobial infections were excluded from the study.

### 2.2. Laboratory Investigations

Ascitic fluid cell counts were determined by automated cell blood counter. Specimens were cultured on blood agar mediums. The basal agar mediums were autoclaved and then cooled to the 50 degrees of Celsius in the laboratory environment. After then, defibrinated blood (5–7%) was added to the basal mediums. Prepared blood agar medium was transferred to the sterile plates in order to culture the obtained samples. Hemolysis pattern was visually observed.

### 2.3. Antimicrobial Susceptibility

Antimicrobial susceptibility testing of isolated bacteria was determined according to the guidelines of the National Committee for Clinical Laboratory Standard's Institutes (CLSI) disk diffusion method. Mueller-Hinton agar was the handled medium. A 0.5 McFarland turbidity standard of the bacterial suspension was adjusted using barium sulphate precipitate. Cultured mediums were stored in laboratory environment for 24 hours. After then, the results were evaluated as sensitive or resistant according to the diffusion radiation. Samples with two or more isolated microorganisms suggestive of secondary peritonitis were excluded. Antibiotic disks used for gram-positive and gram-negative strains were as follows: gentamicin, vancomycin, cefalotin, clindamycin, erythromycin, oxacillin, rifampin, cotrimoxazol, ciprofloxacin, amikacin, imipenem, ceftazidime, ceftriaxone, ampicillin sulbactam, and piperacillin. Antibiotic disks were obtained from HiMedia brand, India, Pakistan, and/or Iran.

This study was carried out in accordance with the principles of the Declaration of Helsinki and was formally approved by the Institutional Ethical Committee.

### 2.4. Statistical Analysis

The SPSS software v.16 for Windows (Chicago, Illinois, USA) was used for analysis. Variables were described as mean (standard deviation, SD) or proportion. Student's *t-*test and chi-squared test were used for comparison of the continuous and categorical variables, respectively. Odd ratios (ORs) were derived by cross tabulating the number of favorable conditions. A two-tailed *P* value < 0.05 was considered statistically significant.

## 3. Results

The mean age (SD) of the study population is 51 (±9) and the male: female ratio is 1.25. Of the total of 1981 ascitic fluid samples, 482 samples (24.33%) were diagnosed as SBP. Out of these samples, 314 samples (65.15%) were identified as culture-positive SBP and 168 samples (34.85%) were culture-negative neutrocytic ascites. Males significantly had higher prevalence of positive culture (OR (95% CI) = 2.69 (1.08–3.06), *P* < 0.01).

Overall, the causative microorganisms of culture-positive episodes of SBP were mainly gram-negative organisms (62.9%). Gram-positive and nonbacterial organisms were responsible for 28.8% and 8.3% of the culture-positive samples. Of 228 episodes of bacterial SBP, 81.25% were owing to anaerobic fecal bacteria and 18.75% were aerobic.

Table 1 represents different microorganisms isolated from ascitic samples regarding different wards of the hospital.* E. coli* was the most prevalent causative microorganism isolated in all wards. As a whole, 16% of the culture-positive episodes of SBP were owing to skin contamination including the Coagulase-negative* Staphylococcus epidermidis* (6.7%), fungal species (8.3%), and* Stenotrophomonas maltophilia* (1%).

Table 2 represents the antibiotic resistance pattern of the isolated organisms. Overall, resistance to ciprofloxacin and ofloxacin was found in 54.6% and 57.1% of the isolated organisms. However, organisms were most sensitive to vancomycin, chloramphenicol, imipenem, piperacillin-tazobactam, tazobactam, meropenem, gentamicin, and amikacin.

In order to assess the trend of antibiotic resistance over time, samples were dichotomized into three-year periods (Table 3). There were no significant differences in the proportion of culture-positive organism as well as the frequency of gram-negative and gram-positive strains in this period.

Additionally, considering these two time periods, the overall antibiotic resistance rates to cefotaxime (62.5%, 85.7%), ciprofloxacin (30, 59.8%), amikacin (19.8%, 29%), ceftazidime (73%, 82.1%), ofloxacin (36.8%, 50%), and oxacillin (35%, 51.6%) were increased (*P* < 0.01 for all). However, no changes in rate of ceftriaxone resistant strains were observed (57.8%, 59%, *P* = 0.12).

## 4. Discussion

There is an apparent lack of data on current spectrum of causative microorganisms of SBP and their antibiotic sensitivity in our region. Herein, the frequency and the patterns of antibiotic resistance among isolated microorganisms from ascitic fluid samples were determined using data collected over six years.

In this study, 24.33% of samples were diagnosed as SBP from which 65.15% were culture positive. The remaining 34.85% were considered as culture-negative neutrocytic ascites. * *The culture-negative neutrocytic ascites have been estimated to occur in 30 to 60% of patients with SBP [1, 10]. This could also be the result of poor culturing techniques or late-stage resolving infection [10]. Nonetheless, empirical antibiotic therapy should be initiated in all patients with PMNL ≥ 250 cells/mm^3^ [1, 10].

Historically, gram-negative bacteria were known as the most prevalent cause of culture-positive samples [2, 3, 7]. However, the etiological pattern of peritonitis varied in different geographical regions [7, 11]. In the present study gram-negative bacteria are among the main etiological agents (62.9%) isolated from ascitic fluid samples and gram-positive bacteria (28.8%) are the next. Our data also indicated that no significant changes in the proportion of gram-negative to gram-positive infections occurred during these 6 years. Although this pattern still holds true in some countries, recent studies suggested an increasing trend in infections caused by gram-positive cocci [8, 9, 12]. * *In the present study, the most prevalent isolated organisms in a descending order were as follows:* E. coli* (33.8%),* Staphylococcus aureus* (8.9%),* Enterococcus* (8.6%),* Acinetobacter* (8%),* Candida* (7.3%),* Staphylococcus epidermidis* (6.7%), and* Klebsiella* (5.4%). Our result indicated that* E. coli* is still the most common cause of culture-positive SBP, independent of the wards. This corresponds to the data obtained in other investigations [1–3, 7, 11, 13–15].

On the other hand, recent studies suggest an increase in the prevalence of enterococcal SBP [8, 9]. In a 12-year retrospective study in Germany, the frequency of enterococcal infections was increased from 11% to 35% and associated with increased resistance to cephalosporins [16]. Other investigations unveiled the poor prognosis of enterococcal SBP and declared that* Enterococcus* strains were mostly resistant to third-generation cephalosporins range between 77% and 100% [8, 17]. As can be seen, ceftriaxone and ceftazidime were inactive against 100%* Enterococcus* strains in this study. Our results were also consistent with those obtained in Korea indicating that enterococcal SBP was susceptible to ampicillin-gentamicin as well as vancomycin [8].

Furthermore,* Streptococcus pneumoniae* was isolated from 0.6% of ascitic samples, which was noticeably lower than some other reports [7, 11]. This could be explained by implementing pneumococcal vaccination in patients with cirrhosis. * *The current guideline [1, 2] recommended initiating empirical antibiotic treatment following the diagnosis of SBP. Since the most frequently isolated microorganisms were gram-negative enteric bacteria, third-generation cephalosporins are suggested as the first-line therapy for SBP. Amoxicillin-clavulanic acid and quinolones (ciprofloxacin, ofloxacin) were also known as effective alternatives [1, 2].

Besides, the antibiotic resistance rates could vary in different region based on the pattern of antibiotic consumption. A number of studies were conducted in different countries to assess the efficacy of the current guideline and help the clinicians to choose the most appropriate antibiotic as first-line treatment. Recent studies notice the emergence of resistance to third-generation cephalosporins. The rates of cephalosporin resistance in patients with SBP were shown to be 21% to 45% [2, 7, 15, 16, 18, 19]. The exposure to systemic antibiotics and nosocomial infections was introduced as independent predictors of resistance to first-line antibiotic regimens [19, 20]. On the contrary, study conducted in Korea declared that cefotaxime could still be the choice of primary empirical antibiotics for the treatment of SBP [7]. Another study in Spain indicated that a short course of ceftriaxone is efficient for resolution of 73% of patients [12].

In this study, overall antibiotic resistance to third-generation cephalosporins and quinolones was as follows: cefotaxime and ceftazidime (77.3%), cefoxitin and cefixime (71.3%), ceftizoxime (70%), cefazolin (61.9%), ceftriaxone (58.2%), ciprofloxacin (45.4%), oxacillin (44%), and ofloxacin (42.9%). In addition, about 71.3% of strains were resistant to coamoxiclav.

Recent study in India also indicated a low response rate to third-generation cephalosporins in patients with SBP. As the sensitivity rates to ceftriaxone were 50%, they suggest that cefoperazone-sulbactam could be a better alternative choice [15]. In the present study,* E. coli*, the predominant isolated pathogen, was most sensitive to imipenem, piperacillin-tazobactam, amikacin, ceftizoxime, and gentamicin, whereas only 20–30% of* E. coli* isolates were sensitive to cefotaxime and ceftazidime and the sensitivity rates to ceftriaxone were 43.4%.

To identify the occurring changes in antibiotic resistance rates of the causative agents, we divided the study period into two 3-year intervals. The resistance rate to cefotaxime, ceftazidime, ciprofloxacin, amikacin, and oxacillin was significantly increased during this time period.

Overall, results of the present study highlight the emergence of resistant strains in our region, as most of the isolated bacteria showed an increased level of resistance to first-line empirical antibiotics. Besides, our rates of cephalosporins resistance are noticeably higher than most of the published literature [2, 7, 14, 18, 19]. Higher resistance rate to the third-generation cephalosporins in this study may be explained by indiscriminate use of these antibiotics during the past decade in our region. In contrary, most of the isolated organisms were sensitive to imipenem, piperacillin-tazobactam, and gentamicin. This may be due to the less frequency of usage of these drugs, as they were usually prescribed in complicated patients.

Precise knowledge about previous SBPs, prior history of antibiotic consumption, and/or use of SBP prophylaxis would be beneficial in explaining the results. Unfortunately, there was little information in this regard in our retrospective database. Nevertheless, as there have been no reports clearly assessing the microbial agents and antibiotic resistance of SBP in our region, our study still declares its critical role in elucidating the situation for the first time in the region.

This study suggests that the current recommended empirical antibiotics need to be reassessed. The empirical treatment of SBP should be adapted to the local epidemiological pattern of antibiotic susceptibility, in order to decrease the morbidity and mortality associated with SBP.

## 5. Conclusion

Present study indicates that, during 2005–2011 time period, the microbial etiology of SBP remains relatively constant; however, the antibiotic resistance rate especially for third-generation cephalosporins (including cefotaxime and ceftazidime), ciprofloxacin, and ofloxacin increased dramatically. Congruent with these findings, only 10–20% of strains were sensitive to cefotaxime and ceftazidime. This pattern must be watched closely and taken into account in empirical antibiotic treatment.

## Figures and Tables

**Table 1:** Profiles of the isolated microorganisms in spontaneous bacterial peritonitis in different wards.

	All	Different wards of the hospital				
		Emergency ward	Internal ward	Surgery ward	ICU ward	Paediatric ward
	*N* (%)	*N* (%)	*N* (%)	*N* (%)	*N* (%)
Positive growth	314 (15.85%)	121 (6.10%)	79 (4.00%)	58 (2.90%)	55 (2.80%)	1 (0.05%)
Organism						
*E. coli *	106 (33.8%)	50 (5.1%)	20 (3.2%)	20 (9.3%)	15 (9.4%)	1 (16.7%)
*Staphylococcus aureus *	28 (8.9%)	14 (1.4%)	7 (1.1%)	7 (3.3%)	—	—
*Enterococcus *	27 (8.6%)	6 (0.6%)	12 (1.9%)	4 (1.9%)	—	—
*Acinetobacter *	25 (8%)	5 (0.5%)	7 (1.1%)	3 (1.4%)	10 (6.2%)	—
*Candida *	23 (7.3%)	8 (0.8%)	6 (1%)	5 (2.3%)	4 (2.5%)	—
*Staphylococcus epidermidis *	21 (6.7%)	8 (0.8%)	8 (1.3%)	3 (1.4%)	2 (1%)	—
*Klebsiella *	17 (5.4%)	7 (0.7%)	3 (0.5%)	6 (2.8%)	1 (0.6%)	—
*Citrobacter *	16 (5.1%)	6 (0.6%)	2 (0.3%)	1 (0.5%)	7 (4.4%)	—
*Pseudomonas *	15 (4.8%)	2 (0.2%)	5 (0.8%)	4 (1.9%)	4 (2.5%)	—
*Enterobacter *	11 (3.3%)	1 (0.1%)	3 (0.5%)	3 (1.4%)	4 (2.5%)	—
Nonhemolytic *Streptococcus *	5 (1.6%)	2 (0.2%)	2 (0.3%)	—	1 (0.6%)	—
*Alcaligenes* sp.	4 (1.3%)	4 (0.4%)	—	—	—	—
Hemolytic Streptococcus	4 (1.3%)	2 (0.2%)	1 (0.2%)	—	1 (0.6%)	—
*Streptococcus * group D	4 (1.3%)	4 (0.4%)	—	—	—	—
*Proteus *	3 (1%)	—	2 (0.3%)	1 (0.5%)	—	—
*Stenotrophomonas maltophilia *	3 (1%)	1 (0.1%)	1 (0.2%)	1 (0.5%)	—	—
*Streptococcus pneumoniae *	2 (0.6%)	1 (0.1%)	—	—	1 (0.6%)	—

**Table 2:** Patterns of antibiotic resistance among isolated microorganisms.

	All positive culture	*E. coli *	*Staphylococcus aureus *	*Enterococcus *	*Acinetobacter *	*Staphylococcus epidermidis *	*Klebsiella *	*Citrobacter *	*Enterobacter *	*Pseudomonas *
Resistance rate %										
Amikacin	17.9%	7.2%	42.9%	50.0%	52.6%	0%	12.5%	22.2%	14.3%	—
Amoxicillin	64.7%	—	—	25%	—	—	—	—	—	—
Ampicillin	45.4%	37.5%	25%	46.7%	—	50%	—	—	—	—
Ampicillin sulbactam	66.6%	50%	—	50%	25%	—	44.4%	27.3%	62.5%	100%
Cefazolin	61.9%	—	33.3%	—	—	16.7%	—	0%	—	—
Cefepime	63.6%	70%	—	—	100%	—	60%		0%	60%
Cefixime	71.3%	67.4%	100%	—	91.7%	—	40%	33.3%	40%	100%
Cefoxitin		—	—	—	100%	20%	—	—	—	—
Cefotaxim	77.3%	72.7%	100%	—	100%	33.3%	66.7%	—	—	100%
Cefoxitin	71.3%	—	45.5%	—	—	100%	—	—	—	—
Ceftazidime	77.3%	75.8%	—	100%	84.6%	—	77.8%	50%	0%	87.5%
Ceftizoxime	70%	8.3%	—	—	4%	—	50%	0%	—	100%
Ceftriaxon	58.2%	56.6%	—	100%	77.8%	—	45.5%	41.7%	66.7%	80%
Chloramphenicol	6.2%	—	33.3%	0%	—	—	—	—	—	—
Ciprofloxacin	45.4%	54.2%	26.3%	42.9%	95%	—	28.6%	44.4%	0%	0%
Clindamycin	50%	0%	44%	100%	100%	—	—	50%	—	—
Cloxacillin	77.8%	—	33.3%	—	100%	—	—	—	—	—
Coamoxiclav	71.3%	—	—	—	100%	—	—	—	—	—
Cotrimoxazol	61.9%	65.9%	28%	61.1%	91.3%	42.9%	77.8%	50%	12.5%	92.3%
Erythromycin	72.7%	—	39.1%	28.6%	—	31.6%	—	0%	—	—
Gentamycin	4.1%	15.6%	54.5%	25%	50%	16.7%	0%	—	33.3%	25%
Imipenem	5.2%	1%	0%	0%	17.4%	0%	0%	0%	0%	13.3%
Ofloxacin	42.9%	—	—	50%	—	—	—	—	—	—
Oxacillin	44%	—	40%	83.3%	—	26.7%	—	50%	—	—
Piperacillin	23.2%	74.4%	0%	—	77.8%	55.6%	—	—	0%	66.7%
Piperacillin-tazobactam	4%	3.7%	0%	—	0%	—	12.5%	0%	0%	0%
Rifampicin	32.4%	—	—	50%	—	14.3%	—	—	—	—
Teicoplanin	63.3%	—	—	50%	—	—	—	—	—	—
Ticarcillin	55.7%	67.5%	—	—	88.9%	—	28.6%	40%	14.3%	20%
Vancomycin	6.7%	—	—	25%	—	0%		0%	0%	—

**Table 3:** Changes in the pattern of causative microorganism and the resistance rate to the first-line recommended antibiotics (2005–2011).

	2005 to 2008	2008 to 2011
Culture positive	15.4%	16.3%
Gram negative	60.2%	64.9%
Gram positive	39.8%	35.1%

Antibiotic resistance rate %		
Cefotaxime	62.5%	85.7%*
Ceftazidime	73.0%	82.1%*
Ceftriaxone	57.8%	59.0%
Ciprofloxacin	30.0%	59.8%*
Ofloxacin	36.8%	50.0%*
Oxacillin	35%	51.6%*

*P < 0.01.
